# Editorial: Novel preclinical model, biomarker, treatment and drug delivery to address immune evasion in cancer

**DOI:** 10.3389/fimmu.2024.1486819

**Published:** 2024-09-13

**Authors:** Chun-Wai Mai, Zuzana Macek Jilkova, Kamal Dua, Bey-Hing Goh

**Affiliations:** ^1^ Faculty of Pharmaceutical Sciences, UCSI University, Kuala Lumpur, Malaysia; ^2^ Univ. Grenoble Alpes, INSERM U 1209, CNRS UMR 5309, Institute for Advanced Biosciences, Grenoble, France; ^3^ Hepato-Gastroenterology and Digestive Oncology, Department, University Hospital Grenoble, Grenoble, France; ^4^ Discipline of Pharmacy, Graduate School of Health, University of Technology Sydney, Ultimo, NSW, Australia; ^5^ Faculty of Health, Australian Research Centre in Complementary and Integrative Medicine, University of Technology Sydney, Ultimo, NSW, Australia; ^6^ Sunway Biofunctional Molecules Discovery Centre, School of Medical and Life Sciences, Sunway University Malaysia, Bandar Sunway, Selangor Darul Ehsan, Malaysia; ^7^ Biofunctional Molecule Exploratory Research Group, School of Pharmacy, Monash University Malaysia, Bandar Sunway, Selangor Darul Ehsan, Malaysia

**Keywords:** cancer immunology, cancer, modeling, biomarker, treatment

Immune cells play a critical role in the defense mechanisms for human health. However, they can also catalyze tumorigenesis, and cancer progression (1). Consequently, cancer cells can escape detection by immune cells, leading to disease advancement and treatment failure (2, 3). In many cancer types, clinical decisions can no longer rely solely on histopathological criteria due to the complexity of cancer-immune cells interaction (2, 4). To improve cancer patient outcomes, it is essential to translate the evidence of immune evasion into clinical practice (1). Precision medicine can only reach its full potential in cancer treatment when disease subtypes are clearly defined through predictive models and supported by comprehensive molecular and immune characterization (1, 5).

To close the knowledge gaps in immune evasion of cancers, the journal launched a Research Topic on “Novel Preclinical Model, Biomarker, Treatment and Drug Delivery to Address Immune Evasion in Cancer” in 2023, resulting in the acceptance of 11 papers for publication. In this editorial, we will highlight the latest findings from these papers and illustrate the proposed way forward in order to target immune evasions in cancer.

In light of the lack of predictive biomarkers for cancer immune evasion, several research teams have proposed potential biomarkers associated with immune evaded cancer. Jacenik et al. from University of Lodz (Poland), University of Utah (USA) and University of Kentucky (USA) identified mitogen-activated protein kinase-activated protein kinase 2 (MK2) as a driver of pancreatic and colon cancer progression by preventing cytotoxic CD8^+^ T cell response. Additionally, Ibrahim et al. from Tokai University School of Medicine, Tohoku University Graduate School of Medicine, and Institute of Biomedical Research and Innovation, Foundation for Biomedical Research and Innovation at Kobe, Japan discovered that the increased level of plasminogen activator inhibitor-1 (PAI-1) contributed to immune evasion in tumor by increasing programmed cell death ligand 1 (PD-L1) expression. The team also observed PAI-1 suppressed T-cell mediated cancer immunity, suggesting a role of PAI-1 in immune evasion. Separately, Cheng et al. from the First Affiliated Hospital of Bengbu Medical College, China, proposed membrane palmitoylated protein 6 (MPP6) as an immune evasion biomarker in hepatocellular carcinoma (HCC). The team further validated the role of *MPP6* using cell lines and tissues, concluding HCC patients with elevated *MPP6* expression exhibit enhanced immune evasion, which is associated with more severe clinicopathological features and poorer survival outcomes. Separately, Zhou et al. from Suzhou Medical College of Soochow University, China and Kunshan Hospital of Jiangsu University, China proposed that low expression of intercellular adhesion molecule 1 (ICAM1) could drive immune evasion among the triple negative breast cancer (TNBC). TNBC patients with low ICAM1 were associated with lower progression free survival (PFS) and a reduced level of immune cells regulation compared to those with high ICAM1 expression. Lower ICAM1 expression was also linked to an increased T cell exhaustion and polarization to M2-type macrophages, leading to cancer progression.

To facilitate the translation of cancer immune evasion findings to clinical practice, the predictive algorithm or model based on immune evasion biomarkers should be developed to support clinical diagnosis. Li et al. from Naval Medical University, Shanghai, China proposed a cancer associated fibroblasts related genes (CAFRG) based signature to assess prognosis, immune characteristics and treatment response for breast cancer patients by combining bulk sequencing and single-cell sequencing findings. Also, Guo et al. from Hebei Normal University, China proposed unfolded protein response (UPR) based signature to classify the prognosis of HCC patients. *ATF4*, *GOSR2*, *PDIA6* and *SRPRB* were upregulated in HCC and higher among the metastatic samples, indicating its role in immune evasion. Furthermore, Chen et al. from First Affiliated Hospital of Chongqing Medical University and Second Affiliated Hospital of Chongqing Medical University, China proposed another HCC diagnostic model using disulfidptosis-related amino acid metabolism genes (DRAGs). The research team demonstrated DRAGs stratified patients with higher risk of disease progression was associated with a stronger immunosuppressive microenvironment and lower immune scores. As a results, DRAGs signature based model could assess the prognosis of HCC patients accurately. Separately, Wang et al. proposed a novel lysosomal signature to construct a predictive model for gastric cancer patients. Eight lysosome-related genes (*ADRB2*, *KCNE2*, *MYO7A*, *IFI30*, *LAMP3*, *TPP1*, *HPS4*, and *NEU4*) were selected to combine with machine learning algorithm to construct an AI-based diagnostic predictor using these lysosome-related genes. Such models could potentially revolutionize future cancer diagnosis and cancer treatment by accurate predict the degree of immune evasion in every cancer patient.

To maximize the benefits of immunotherapy for cancer patients, Ma et al. performed a meta-analysis to identify the risk factors associated with prognosis of HCC patients when undergoing treatment with immune checkpoint inhibitors. From the 47 studies and data collected from >7600 patients, the authors proposed alpha-fetoprotein (AFP), albumin-bilirubin score (ALBI), neutrophil-to-lymphocyte ratio (NLR), Eastern Cooperative Oncology Group (ECOG) performance status, Child-Pugh stage, Barcelona Clinic Liver Cancer (BCLC) stage, tumor number, and vascular invasion as predictors associated with overall survival (OS) and PFS, while combination therapy was only associated with OS prediction model. The models were validated with two independent cohorts of patients. Both OS an PFS models shown good performance and thus, it can be used to predict risk factors associated with prognosis among HCC patients when started on immune checkpoint inhibitors. Independently, Puyalto et al. proposed [^89^Zr]-anti-program cell death protein (PD)-1 immuno-positron-emission tomography (PET) as a promising tool to predict the efficacy of PD-1/PD-L1 inhibitors for non-small cell lung cancer (NSCLC) patients. The [^89^Zr]-anti PD-1 signal correlated with immune cells infiltration, and its signal was higher among the anti-PD-1-responding mice. Similarly, Chai et al. also proposed a non-invasive radiomic model to predict immune cells infiltration among NSCLC patients treated with immune checkpoint inhibitors. Biomarkers from tumor immune microenvironment were used to develop this noninvasive radiomic model to predict the efficacy of immunotherapy, aiming to improve patient’s treatment outcomes.

This collection of articles provides a better understanding of immune evasion in cancers and proposes several translatable models to predict patient’s prognosis and treatment outcomes. We anticipate that future research will further elucidate the complex interaction between immune cells and cancer, leading to a better cancer diagnosis and treatment outcome.
